# Effect of lyophilization on HRP–antibody conjugation: an enhanced antibody labeling technology

**DOI:** 10.1186/s13104-018-3688-8

**Published:** 2018-08-17

**Authors:** Saikant Regidi, Shilpa Ravindran, Ashitha L. Vijayan, Vani Maya, Lakshmi Sreedharan, Jeslin Varghese, Kartik Ramaswami, Manoj Gopi

**Affiliations:** Diagnostic Products Division & Cell Culture Facility, Corporate R&D Division, HLL Lifecare Limited, Akkulam, Thiruvananthapuram, India

**Keywords:** Conjugation, Horseradish peroxidase, ELISA, Immunoassay

## Abstract

**Objective:**

Immunoassay usually deal with the antibody labeling with various reporter molecules, one such useful reporter molecule is horseradish peroxidase (HRPO). Conjugating enzyme with antibody without losing its enzymatic activity is a challenging task. Our aim is to modify existing classical method of conjugating antibodies with HRP to enhance immunoassay techniques with better sensitivity. We used chemicals such as sodium meta periodate to generate aldehyde group by oxidation of carbohydrate moieties on HRPO. The activated form of HRPO is lyophilized and then mixed with 1 mg/ml concentration of antibodies to be conjugate.

**Results:**

After confirming chemical modification of conjugates via UV-Spec and SDS-PAGE independent molecules were used for conjugation and HRP–antibody conjugate. Finally, enzymatic activity of HRP–antibody conjugate was confirmed by performing direct ELISA. Functional properties were analyzed using ELISA with dilution of 1:5000, whereas the conjugate prepared by existing method of conjugation worked with as low dilution of 1:25 with a *p* value highly significant (< 0.001) for classical verses modified method of conjugation preparation. Collectively, this study showed the enhanced ability of antibody to bind more number of HRPO with an additional step of lyophilization in the regular conjugation protocol. Future exploration are necessary on wide range of IgG antibodies.

**Electronic supplementary material:**

The online version of this article (10.1186/s13104-018-3688-8) contains supplementary material, which is available to authorized users.

## Introduction

During 70’s and 80’s diagnosis of a disease largely depended on biochemical and physical examination. The emerging technologies in the field of immunology brought forward the use of labeling enzymes as reporter molecules for the enzyme linked immunosorbent assays (ELISA). Labeled antibodies were extensively used for various immunological applications. The process of labeling involved the formation of a stable, covalent linkage between enzyme and the antibodies [1, 2]. Periodate method was the most common used chemical in conjugation, apart from the periodate various other chemicals like glutaraldehyde, maleimide, 1-ethyl-3-[3-dimethylaminopropyl] (EDC) were also used. These chemicals functions as homomers, heterodimers such as linking molecules applied to conjugate enzyme molecules to antibodies without losing functionality [3–5]. Previous reports proved that the efficiency of conjugate varied widely with the conjugation methods [6, 7]. An ideal coupling procedure should provide maximum yield of conjugate product [8]. There are various reporter molecules existing for the use of antibody labeling such as horse radish peroxidase (HRPO), alkaline phosphatase (ALP) and β-d Galctosidase.

However, HRPO labeled antibody is extensively used for immunological applications due to its structural features, availability and stability. HRPO is a heme glycoprotein of 44 kDa containing 18% of carbohydrate contents surrounding a protein core. Since it is a plant protein, it does not have potentially interfering autoantibodies in the biological samples [9, 10]. Generation of aldehyde group by periodate oxidation of carbohydrate moieties on HRPO is the crucial step in the conjugation of antibodies. These aldehydes formed will combine with the amino group of antibody to form Schiff’s base by reduction using sodium cyanoborohydride, which is very stable [11]. HRPO conjugate preparation to conjugate monoclonal antibodies need to have higher dilution factor to achieve signal amplification in immunological applications to detect pathogens. Therefore, the present study aimed to develop an improved protocol which can increase the yield of the conjugated antibodies and thereby causing reduction in the use of conjugated antibodies for signal amplification in immunoassay applications.

## Main text

### Materials and methods

#### Monoclonal antibodies (mAb) and recombinant antigens

Purified mAb’s and antigens of dengue were procured from Biogate laboratories, Canada (Cat No. DN1-9, AG 02-01-4, AG 02-01-5, AG 02-01-3, AG 02-01-6). These purified antibodies and antigens were used for the labeling of enzymes for immunoassays.

#### Enzymes, chemicals and consumables

Horseradish peroxidase (HRP) was purchased from Sigma Aldrich (Cat. No. P6782) and was stored at 2–8 °C until use. The substrate used for HRP was 3,3′,5,5′-Tetramethylbenzidine (TMB) purchased from Bangalore Genei (Cat. No.62161018010A). Sodium metaperiodate (Cat. No. S1878) and Sodium cyanoborohydride (Cat. No.156159) were purchased from Sigma Aldrich (United States). Dialysis membrane (4 Spectra/Por; Reorder No. 132700) used for desalting purposes was purchased from Spectrum Laboratories (United States) and dialysis tubing closures (LA 404), coomassie brilliant blue R-250 (MB 153) from Hi-media Laboratories (India). ELISA high binding plates (EP2-20X10NO) were purchased from Hi-media (India). All other general chemicals and consumables used for preparation of the buffers and reagents were procured from Sigma Aldrich and Hi-media.

#### Enhanced labeling procedure

Labeling of HRPO was done using the modified version of classical periodate method by incorporating the process of lyophilization in-order to obtain better sensitivity [1]. The process of conjugation involved two steps; adoption of classical method from the periodate method. The step one involves the activation of HRPO was done using 0.15 M Sodium metaperiodate. The activated HRPO was then desalted by using dialysis method with 1× phosphate buffered saline (PBS) for 3 h at Room temperature. Post dialysis, the HRPO was frozen at − 80 °C (New Brunswick) for 5–6 h. The step two involves the overnight lyophilization of frozen HRPO. After this process, 1:4 molar ratios of antibody to HRP taken for the conjugation process. Where stock concentration of antibody were 7.78 mg/ml diluted to 1 mg/ml concentration for conjugation experiment and mixed with previously lyophilized HRPO and incubated at 37 °C for 1 h in a thermo mixer comfort (Eppendorf). Finally, to the conjugate Schiff’s base reaction carried out by adding 1/10th volume of sodium cyanoborohydride and then incubated at 4 °C for another 2 h (Additional file 1: Figure S1). The conjugate thus formed was kept for overnight dialysis against 1× PBS in room temperature. Any commercially available stabilizers can be added to the conjugate for long-term stability. This conjugate can be stored at 4 °C for 6 months and at − 20 °C for long-term storage.

#### Sodium dodecyl sulfate polyacrylamide gel electrophoresis (SDS-PAGE)

SDS-PAGE experiment performed based on previously reported method by Green and Sambrook [12]; 10% resolving gel was prepared using recommended volumes of SDS-PAGE components and 1 ml butanol was overlaid to form a uniformly layered gel. After polymerization, the gel was washed several times with distilled water to remove traces of un-polymerized acrylamide and butanol. The stacking gel was prepared and poured it over the polymerized resolving gel. The samples were then mixed with sample buffer containing β-mercaptoethanol and heated at 95 °C for 10 min for reducing and for non-reducing samples are mixed with sample buffer alone preceding to load on to the gel. A total volume of 10 µl of samples were mixed 2 µl sample buffer to load on to the gel along with prestained protein Ladder (MBT092 from Hi-media). An initial voltage of 85 V applied to the gel and after the dye front moved into the resolving gel, the voltage gradually increased to 120 V. The gel was run until the dye front reaches the bottom of the resolving gel. The gel was washed with distilled water and stained using coomassie brilliant blue (CBB) staining solution for 1 h. The stained gel was washed using distilled water and soaked in de-staining solution for 2–4 h. The gel image was photographed using Bio-Rad gel documentation system (Gel Doc™ EZ System #1708270).

#### Ultraviolet and visible (UV–Vis) spectrophotometer analysis

A Shimadzu UV-Pharmaspec 1700 recording double beam UV–visible spectrophotometer with data processing system was used. UV spectra of reference and sample solutions were recorded in 10-mm quartz cells (Hellma Analytics, Art. No. 115-10-40) at a wavelength scanning speed of approx. 3000–10 nm/min. The concentration of conjugated and control in its solutions were determined in between in wavelength ranging from 280 to 800 nm.

#### Enzyme linked immunosorbent assay (ELISA)

Direct ELISA was performed using the conjugates prepared by the enhanced technique. 100 µl of antigen of 100 ng per well concentration was coated on to the high binding uncoated ELISA plate using carbonate buffer (pH 9.2) followed by overnight incubation at 4 °C. The plates were then washed using PBST buffer and blocked with 2% skim milk powder for 1 h at 37 °C. The conjugate was serially diluted from 1:100 onwards in 1× PBS containing 2% bovine serum albumin (BSA) and added to the antigen coated plate and incubated at 37 °C for 1 h in dark. Plates were washed using PBST followed by taping on dry tissue paper to remove completely any trance of solution and TMB substrate was added and further incubated for 20 min in dark. Reaction was stopped by adding stop solution and the absorbance was read at 450 nm using Tecan Infinte 200 Pro ELISA reader. Similar concentration of antigen and ELISA protocol was used for comparison purpose conjugate prepared by the existing classical method.

To study sensitivity of the dengue antibody–HRP conjugates we have performed direct ELISA by coating different concentrations of dengue antigen starting from 100 to 1.5 ng. Followed by blocking as suggested above protocol and 100 µl of conjugate preparation with dilution factor 1:1000 was added to each well. Followed by rest of the protocol for ELISA same as above-mentioned and absorbance read to 450 nm.

#### Statistical analysis

The statistical analysis of the assays were done by plotting dilution response curve with Graph Pad software (Graph pad instat 3.1 version) using the secondary antibody conjugates prepared by both the methods. Turkey Kramer multiple comparison tests expressed as mean of three independent experiments. Statistical analysis was done using one-way annova followed by graph pad software to find out the significance of the results obtained. p value < 0.001 was considered to be statistically significant.

### Results

**Fig. 1:**
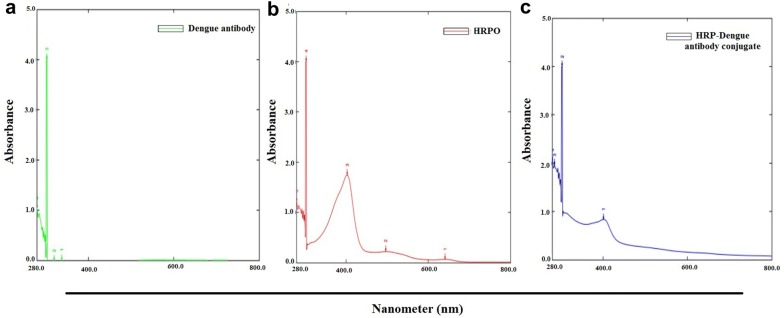
Image of UV–vis absorption spectra **a** antibody (green), **b** Horseradish peroxidase HRPO (red) and **c** HRP–antibody conjugate (blue) in the suspension solution

The conjugate obtained by the modified method of existing classical protocol was taken for further experiments. The initial confirmation of conjugation was done by UV spectrophotometer and SDS-PAGE analysis. In UV-spec the wavelength scan of the conjugate was performed at a range of 280–800 nm.The result obtained was then compared with the results of unconjugated HRPO and unconjugated antibody alone. The observations showed that HRPO gave a peak at 430 nm and antibody at 280 nm. Due to the modification of HRPO during the conjugation procedure, there was a shift in the absorption which resulted in a small peak at 430 nm when compared to the peak of HRPO alone which confirms the efficient chemical modification leading to conjugation (Fig. 1).

**Fig. 2:**
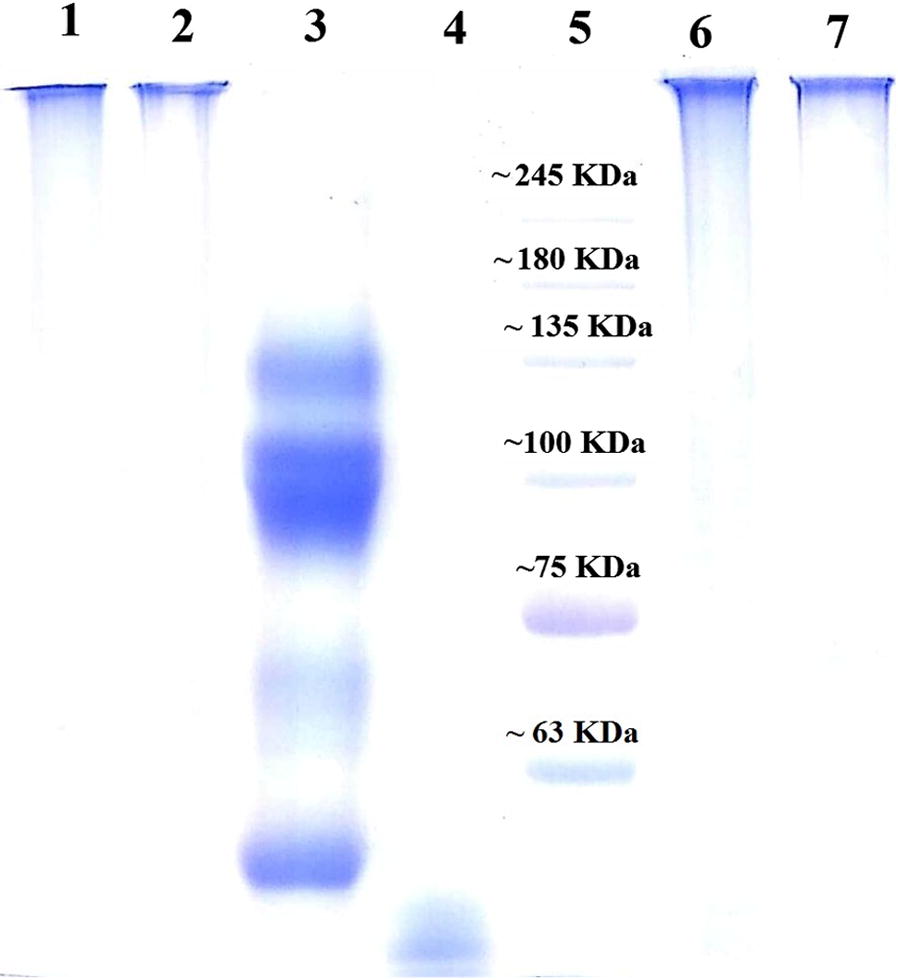
The protein separation pattern on 6% SDS-PAGE gel shows Lane-1: classical method conjugate of antibody–HRP (reduced), Lane-2: modified method conjugate of antibody–HRP (reduced), Lane-3: antibody (reduced), Lane-4: HRP (reduced), Lane-5: protein molecular ladder, Lane-6: classical method conjugate of antibody–HRP (non-reduced), Lane-7: modified method conjugate of antibody–HRP (non-reduced)

In support to the above results, SDS-PAGE was also performed to confirm the conjugation of HRPO to antibodies. Conjugate resulted from both classical and modified method (Lane-1 and 2) are treated along with HRPO and dengue antibodies for heat denaturation at 95 °C, alongside non-reducing conjugates (Lane-6 and 7) prepared and loaded for the comparison. Subsequent staining showed that there was no migration seen in both procedural involved conjugates. Whereas lower molecular size HRPO (Lane-4) reached almost at the end of the gel and antibodies (Lane-3) are denatures and shown mobility according to its molecular size (Fig. 2). This indicated that the conjugation of the antibody to HRPO was done efficiently.

**Fig. 3:**
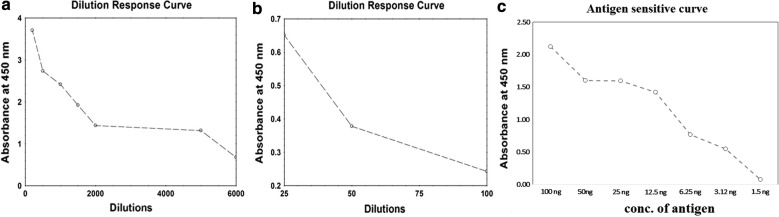
Graphical representation ELISA data displaying classical method verses modified method using dengue labeling antibodies for HRP conjugation. **a** Demonstration ELISA data in terms of dilution factor for modified protocol, **b** demonstration ELISA data in terms of dilution factor for standard protocol and **c** demonstration of antigen standard curve using 1:1000 dilution factor of modified protocol antibody–HRP conjugate

The activity and sensitivity of the conjugate was finally confirmed by performing direct ELISA. Based on the findings from dilution response curve, we found that better sensitive conjugates were formed with enhanced method of conjugation and significant variation was seen in selectivity of ELISA results. In lyophilized method ELISA reading was obtained with dilution of even 1:5000 sensitivity to detect antigen, but in conjugate prepared by using the classical method of conjugations it needed significantly high dilutions of 1:25 for the same amount of antigen preparation. Statistical analysis showed that the p value obtained was found to be highly significant (< 0.001) for all the comparisons made between the values obtained from modified method and the classical method of conjugations as shown in Fig. 3a, b. The same protocol was applied for other antibodies used in our laboratory and similar results were found when preformed using ELISA tests. While antigen standard curve experimentation suggest that conjugate preparation using modified method can detect antigen as low as 1.5 ng (Fig. 3c).

### Discussions

The primary objective of any conjugation method is to produce a stabile conjugate without affecting its antigen binding capability. Modification of carbohydrate moieties of enzyme gives much superior advancement in comparison to other techniques involved in modification of antibodies itself. The conjugation method proposed in the present paper based on classical method of use peridodate reagent and implementing lyophilization after activation of HRP, allows storage of active compound at cold condition for longer duration and enhances sensitivity of antibody due to conjugation of more number of HRP and due to its poly-HRP nature. In conjunction with collision theory, molecules must collide to react and rate of reaction is proportional to number of reacting molecules present in the solution. In term of chemical reaction, number antibody molecules, active HRP molecules and volume of reaction are decisive factors in successful conjugation of antibody to HRP. To enhance the binding capacity of antibodies lyophilized activated HRP made it freeze-dried, which reduced reaction volume without changing the amount of both the reactants. The advantage of the additional step is that active HRP can maintained at 4 °C for longer duration. Evaluation of the validity of prepared conjugates, its sensitivity in detection of antigen-antibodies reaction needs to be further explored. The ELISA was performed with recombinant antigen procured commercially and performance of the ELISA was assessed based on the evaluations of classical methodology vs modified methodology. We observed that modified protocol produced significant increase in the antibody titter than the classical conjugation method, which improves the overall efficiency of the ELISA performance.

### Conclusion

The study result proves that antibody–HRP conjugates overcome the limitation of immunological test like ELISA to detect lower amounts of biomarkers presence in the system. Enhanced sensitivity of HRPO-antibody conjugate using modified periodate methodology boost capability of diagnostic ELISAs in various diseases perspectives, and may ultimately enable early diagnosis with better prognosis. Still a full-scale broader study need to be performed with large number of antibodies to validate viability of the technology developed for upscale in industrial sector. In conclusion, this study demonstrated a simple and stable method for two-step bio-conjugation of protein with carbohydrate moieties consisting reporter molecule for immunoassay purposes.

## Limitation

The main disadvantage is that since the conjugation based on chemical reaction involved carbohydrate moieties modification, one of the protein molecules should processes such modification favoring non-functional moieties. In addition, prior to the antibody conjugation one should make sure removal of any azide stabilizers added to antibody due to presence of amino group it may interface with conjugation protocol and leads to outcome protocol efficiency.

## Additional file

**Additional file 1: Figure S1.** Illustrative representation of chemical reaction takes place at each step of chemical reaction followed by highlights lyophilization step modification to the standard protocol.

## Abbreviations

UV-Spec: ultraviolet–visible spectroscopy
SDS-PAGE: sodium dodecyl sulfate–polyacrylamide gel electrophoresis
EDC: 1-ethyl-3-[3-dimethylaminopropyl]carbodiimide
ELISA: enzyme-linked immunosorbent assay
IgG: immunoglobulin G
CBB: coomassie brilliant blue
PBST: phosphate buffered saline with tween 20
PBS: phosphate buffered saline
BSA: bovine serum albumin
TMB: 3,3′,5,5′-tetramethylbenzidine
