# Supplementary figures and images for: Effect of lyophilization on HRP–antibody conjugation: an enhanced antibody labeling technology
Source: BMC Res Notes. 2018 Aug 17;11:596. doi: 10.1186/s13104-018-3688-8 (PMC6102881; doi:10.1186/s13104-018-3688-8)

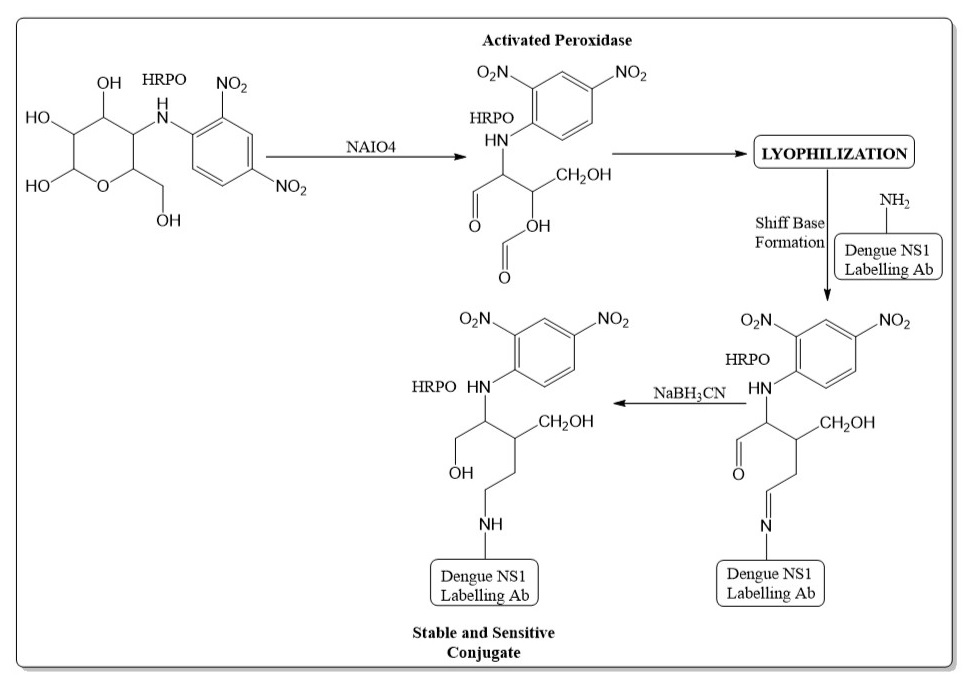

Supplement: Supplementary file 1 — Additional file 1: Figure S1. Illustrative representation of chemical reaction takes place at each step of chemical reaction followed by highlights lyophilization step modification to the standard protocol. [file 13104_2018_3688_MOESM1_ESM.jpg]
